# Health-associated changes of the fecal microbiota in dairy heifer calves during the pre-weaning period

**DOI:** 10.3389/fmicb.2024.1359611

**Published:** 2024-04-26

**Authors:** Sabine Scully, Bernadette Earley, Paul E. Smith, Catherine McAloon, Sinéad M. Waters

**Affiliations:** ^1^Animal and Bioscience Research Department, Animal and Grassland Research and Innovation Centre, Teagasc Grange, Meath, Ireland; ^2^School of Veterinary Medicine, University College Dublin, Dublin, Ireland; ^3^School of Biological and Chemical Sciences, University of Galway, Galway, Ireland

**Keywords:** dairy, calf health, diarrhea, microbiota, colostrum, 16S rRNA sequencing

## Abstract

**Introduction:**

Neonatal calf diarrhea is a multifactorial condition that occurs in early life when calves are particularly susceptible to enteric infection and dysbiosis of the gut microbiome. Good calf health is dependent on successful passive transfer of immunity from the dam through colostrum. There are limited studies on the developing gut microbiota from birth to weaning in calves.

**Methodology:**

Therefore, the objective of this study was to examine the effect of immune status and diarrheal incidence on the development of the fecal microbiota in Jersey (*n* = 22) and Holstein (*n* = 29) heifer calves throughout the pre-weaning period. Calves were hand-fed a colostrum volume equivalent to 8.5% of their birthweight, from either the calf’s dam (*n* = 28) or re-heated mixed colostrum (≤2 cows, ≤1d; *n* = 23) within 2 h of birth. All calves were clinically assessed using a modified Wisconsin–Madison calf health scoring system and rectal temperature at day (d) 0, d7, d21, or disease manifestation (DM) and weaning (d83). Weights were recorded at d0, d21, and d83. Calf blood samples were collected at d7 for the determination of calf serum IgG (sIgG). Fecal samples were obtained at d7, d21/DM [mean d22 (SE 0.70)], and at weaning for 16S rRNA amplicon sequencing of the fecal microbiota. Data were processed in R using *DADA2*; taxonomy was assigned using the SILVA database and further analyzed using Phyloseq and MaAsLin 2.

**Results and discussion:**

Significant amplicon sequence variants (ASVs) and calf performance data underwent a Spearman rank-order correlation test. There was no effect (*p* > 0.05) of colostrum source or calf breed on serum total protein. An effect of calf breed (*p* < 0.05) was observed on sIgG concentrations such that Holstein calves had 6.49 (SE 2.99) mg/ml higher sIgG than Jersey calves. Colostrum source and calf breed had no effect (*p* > 0.05) on health status or the alpha diversity of the fecal microbiota. There was a relationship between health status and time interaction (*p* < 0.001), whereby alpha diversity increased with time; however, diarrheic calves had reduced microbial diversity at DM. No difference (*p* > 0.05) in beta diversity of the microbiota was detected at d7 or d83. At the genus level, 33 ASVs were associated (adj.*p* < 0.05) with health status over the pre-weaning period.

## 1 Introduction

Diarrhea accounts for 40% and 50% of calf mortality from birth to 5 months in Ireland (DAFM, 2021) and internationally (Cho and Yoon, 2014; Chen et al., 2022), respectively. Gastrointestinal (GI) disease in the form of neonatal calf diarrhea is the most common cause of mortality in calves of <1 month of age, with 8.7% of beef and 25.5% of dairy calves affected (Todd et al., 2018). Neonatal calf diarrhea is associated with reduction in average daily gain (ADG), fertility performance, and first lactation milk yield (Abuelo et al., 2021). Additionally, there is a negative effect on animal welfare due to ataxia, dehydration, weakness, and anorexia, causing stress and discomfort to the animal, as well as prolonged suppression of immune function (Carter et al., 2021).

Newborn calves are agammaglobulinemic and rely on passive transfer of immunity (PTI) from the dam through colostrum for their first immune protection (McGee and Earley, 2019). Calves with failure of passive transfer immunity are more susceptible to diseases, such as diarrhea, during the pre-weaning period (Johnston et al., 2016). For successful passive transfer of immunity, calves require good quality colostrum (>50 g/L Immunoglobulin (Ig) G) fed in an adequate amount within 2 hours (h) of life (Lorenz et al., 2011) in a hygienic manner (Carter et al., 2021).

The early life GI microbiome has been linked to enteric infections and neonatal calf diarrhea (Oikonomou et al., 2013; Malmuthuge et al., 2019) as a result of naïve microbial communities present prior to the establishment of a stable community (Chase and Kaushik, 2019). Our understanding of both the temporal establishment and factors influencing the colonization of the lower GIT microbiota has greatly benefitted with the use of next generation sequencing (NGS) techniques, such as 16S ribosomal RNA (rRNA; Júnior and Bittar, 2021). For example, NGS has previously been utilized to investigate the colonization and development of the hindgut microbiome (Malmuthuge and Guan, 2017), factors that influence development (Amin and Seifert, 2021), and disruptions to the microbiome during diarrheal disease (Gomez et al., 2017; Chen et al., 2022; Gomez et al., 2022; Cui et al., 2023).

Microbial colonization of the gut begins shortly after birth (Song et al., 2018), potentially even *in utero* (Guzman et al., 2020; Bi et al., 2021). Development of the gut microbiome follows a regular pattern from colonization to stable community during the pre-weaning period (Du et al., 2023). The GI microbiome once established is comprised of commensal and pathogenic microorganisms (bacteria, archaea, fungi, and viruses) that exist in equilibrium within a healthy host (Gomez et al., 2019). However, instability during colonization leaves the microbiome at risk of dysbiosis, where the microbial community experiences a loss in commensal microbes in conjunction with the proliferation of pathogenic species, resulting in suppressed immune function and increased inflammation at the mucosal layer (Chase and Kaushik, 2019). The hindgut microbiome of the calf in early life changes rapidly (Du et al., 2023), which can quickly result in dysbiosis if harmful microbes outcompete beneficial microbes.

Several factors influence microbial colonization and development in the gut (Gomez et al., 2019) including the dam’s microbiome (Li et al., 2022), colostrum intake (Song et al., 2018), nutrition (diet and feed supplements), age, and stressful life events, such as weaning and exposure to antimicrobials (Amin and Seifert, 2021). Colostral components, including oligosaccharides, are an important source of energy for the naïve microbial communities within the gut, which have been shown to promote beneficial microbial colonization and physiological development of the small intestine (Gomez et al., 2019). In addition, the preparation of colostrum prior to administration to the calf has been shown to influence the microbial colonization of the lower GIT (Song et al., 2019). Other factors such as farm of origin and host genetics have been identified as potentially influencing the development of the lower GIT microbiota, as well as response to diarrheal incidence, in dairy calves (Gomez et al., 2017; Slanzon et al., 2022). However, there is a lack of information, in particular, on the development of the fecal microbiota of calves during the pre-weaning period with a focus on changes before, during, and after recovery from diarrheal disease. Therefore, the objectives of the present study were to examine (1) effect of artificial feeding of colostrum, sourced from either the calves’ dam or a mixed source, on calf passive immunity and (2) effect of health status including diarrheal disease and recovery on the fecal microbiota during the pre-weaning period.

## 2 Materials and methods

### 2.1 Ethics statement

The experiment was conducted at the Dairygold Research Farm in Kilworth, Co. Cork, Ireland (Teagasc, Animal and Grassland Research and Innovation Centre, Moorepark, Fermoy, Co. Cork, Ireland; 52°09′N; 8°16′W). The Teagasc Animal Ethics Committee approved (TAEC2021-327) all animal procedures used in the study, and the study was conducted using procedures consistent with the experimental license (AE19132/P148) issued by the Irish Health Products Regulatory Authority in accordance with European Union legislation (Directive 2010/63/EU) for the protection of animals used for scientific purposes.

### 2.2 Animal management

#### 2.2.1 Dam management

Calves were selected from a single spring calving, multi-parous herd [1–10 lactations, mean 2.83 (SEM 0.33)] consisting of Holstein–Friesian (*n* = 31) and Jersey (*n* = 20) cows. All cows were vaccinated for rotavirus, bovine corona virus, and *E. coli* (r-c Rotavec®Corona, MSD Animal health, Ireland) 4 to 8 weeks prior to calving and received an annual booster for infectious bovine rhinotracheitis (IBR; Rispoval® IBR-Marker inactivated, Zoetis Belgium S.A., Dublin Ireland), bovine viral diarrhea virus (BVD; Bovilis® BVD, Intervet Ireland Limited, Dublin, Ireland), *Leptospirosis* (Bovilis® Leptavoid®-H, Intervet Ireland Limited, Dublin, Ireland), and *Salmonella* (Bovilis® Bovivac S, Intervet Ireland Limited, Dublin, Ireland). Cows calved indoors in a shed away from the main herd, with concrete floors and deep straw bedding.

#### 2.2.2 Colostrum management

Colostrum was obtained from each dam using a portable milking unit (MK100 034904 2017 Inv Ref17027151). Within 2 h of birth, each calf received 8.5% of their BW (in liters: L) of colostrum from either their dam or a refrigerated colostrum source. Refrigerated colostrum consisted of colostrum from two or less cows that had calved within the previous 24 h and was stored in a refrigerator (4°C) and is referred to as “mixed.” Mixed colostrum was heated to approximately 38° C using a water bath prior to feeding. Colostrum quality was tested using a digital brix refractometer (Hanna Digital Refractometer for Sugar, HI9681).

#### 2.2.3 Calf management

Fifty-one spring-born dairy heifer Holstein (HO; *n* = 29, birth weight (BW) 34.7 (SEM 0.69) kg) and Jersey (JE; *n* = 22, BW 25.9 (SEM 0.81) kg) calves were enrolled in this study.

Calves remained with their dam for 30 min after calving. Upon removal, each calf was weighed using a calibrated weighing scale and the navel was disinfected (Foradine 10, povidone iodine 10%). The calf was then moved to an individual pen (1.4 m × 0.46 m) until day (d) 3 after birth. From d3 until weaning, calves were housed in group pens (9.1 m × 4.6 m) with 40 calves of similar age.

Calves received, on average, 2.6 L (SE 0.07) of colostrum within 2 h of birth either from the dam (*n* = 28) or a mixed source (*n* = 23). Calves were then fed 2 L of transition milk twice daily using a bucket with a teat. From d3 to d14, calves received transition milk twice a day using a milk trough with teats, gradually increasing from 4 L to 6 L per calf per day. From second feeding to d7, calves received paromomycin (Parafor™, BIOVET JSC, Bulgaria) preventatively against *Cryptosporidiosis* within each milk feed. At d14, calves were transitioned to an allocation of 6 L of milk replacer [Heiferlac GB/IRL, Volac Feeds LTD, Ireland; 26% crude protein (CP)] per day fed via automated calf feeder (Forster Technik, Volac). Calves had access to roughage (straw) and pelleted concentrates (Sweet start calf starter pencils, 20% CP, Southern Milling LTD., Co. Cork, Ireland) from d3 until weaning.

Calves were housed indoors in deep straw-bedded pens and had access to an outdoor area that was bedded with woodchips. At 2 weeks of age, calves received an oral dose of toltrazuril (Bovicox, KRKA, Slovenia) for Coccidiosis and were vaccinated against *Clostridia* (Tribovax10, Intervet Ireland LTD., Ireland) and respiratory disease pathogens (Pneumovac PLUS, Animal Health Distributors, Ireland) subcutaneously and received booster vaccinations 3 weeks later against the respiratory disease pathogens (Pneumovac PLUS, Animal Health Distributors, Ireland).

Calves were weaned gradually off milk replacer for 7 days based on targeted weights (HO 85 kg; JE 75 kg) and concentrate intake (minimum 1 kg of concentrate per calf per day for 3 consecutive days). Once weaned, calves were turned out to pasture and offered 1 kg of concentrates/calf/day (Super Calf Rearer Nuts, 17% CP, Southern Milling LTD., Ireland).

##### 2.2.3.1 Clinical assessment

Throughout the study, clinical assessment of animals was performed by the same scientist (S. Scully) to minimize sampler bias. Calves were clinically assessed using a modified Wisconsin–Madison calf health scoring system (McGuirk and Peek, 2014) and rectal temperature (RT) was measured at d0-perinatal, 7, 21/DM, and 83 (at weaning) post-birth. The Wisconsin–Madison system classifies RT, presence of cough, appearance of eye and nasal discharges, and ear position with scores ranging from 0 to 3 (from normal to very abnormal). Fecal consistency and navel and joint swelling were additionally scored on a scale of 0 to 3. Dehydration was assessed using a skin tent test, with scores ranging from 0 to 2, as was coat condition. Mucus membranes (lower inner eyelid) were used to assess anemia and scores ranged from 1 to 5 [from normal to very abnormal (severe anemia)]. Diarrheal disease was assessed using fecal scores (0 = normal, 1 = semi-formed, 2 = moderate diarrhea, and 3 = severe diarrhea). Calves that received a fecal score of 2 or higher were observed for 48 h for repeated diarrheal stool before being classified as diarrheic. Health status was defined as calves with diarrhea (diarrheic; *n* = 27) and absence of symptoms as “healthy” (*n* = 24) during the pre-weaning period.

Calves presenting with diarrhea received a binding agent (kaolin powder 15–20 g/feeding) in milk meals and were supplemented with electrolyte fluids (3–4 L per calf) twice a day between milk meals. Where possible, sick calves were moved to individual pens with deep straw bedding and heat lamps. During high diarrheal incidence among calves, sick calves remained in group pens, and all calves in the pen received supportive therapies as outlined above.

### 2.3 Fecal and blood sample collection

**Figure 1:**
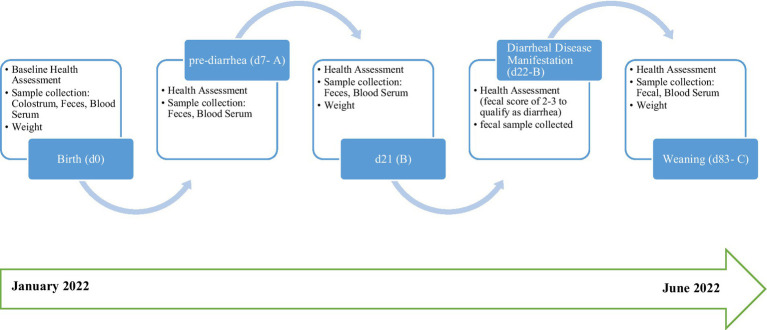
Graphical representation of trial work and sample collection.

Calves were sampled at three pre-determined time-points (d7, 21, and 83 (weaning) post-birth), as shown in Figure 1, where temperatures in calf sheds ranged from 2.3 to 12.5°C during the trial period. Calves were clinically observed daily from birth to weaning for symptoms of diarrheal disease. Calves with diarrhea were clinically assessed, and fecal samples were collected and monitored for 24 to 48 h. Calves were designated as “healthy” or “diarrheic” based on the presence or absence of diarrheal disease symptoms. Fecal samples were collected by rectal stimulation with sterile gloves to facilitate collection at three separate time-points: on d7, d21 or at disease manifestation, and d83 at weaning. Samples were placed in a sterile 25 mL screw cap tube and immediately snap frozen in liquid nitrogen. Samples were then transported to the laboratory on dry ice and stored at −80°C for further analysis. Two samples from the healthy cohort collected on d7 were misplaced, and one sample from the diarrheic cohort was not obtained at d83.

Whole blood was collected from calves at the d7 [mean age d6.88 (SE 0.24)] via jugular venipuncture using an 18 g × 1″ needle [Vacutainer® PrecisionGlide™ Single Sample Veterinary Needle, Becton Dickinson (BD)] inserted into a 1 × 9 mL VACUETTE® TUBE Serum Clot Activator (Greiner Bio-One, Austria). Blood samples were left at room temperature for approximately 90 min after collection and then refrigerated at 4°C until further processing. All blood samples collected were centrifuged at 1,600 × g for 10 min at 20°C. The serum was collected, and aliquots (3 × 1 mL) were transferred to Eppendorf tubes and stored at −80°C until further analysis.

### 2.4 Serum total protein and IgG concentrations

Before laboratory analysis, serum samples collected on d7 were removed from −80°C and allowed to thaw in a refrigerator (4°C) for approximately 24 h. Serum was removed from the refrigerator and stored at room temperature (20°C) for approximately 1 h prior to serum total protein (STP) and radial immunodiffusion (sRID) testing. The quantification of STP was performed by pipetting 100 μL of serum onto the lens of a digital refractometer (DR-303, digital handheld refractometer, Index Instruments LTD, Cambridgeshire, England, United Kingdom), which is similar to the method used by Todd et al. (2018).

Quantification of total IgG concentrations of serum (sIgG) was performed using a commercially available sRID kit (Radial Immunodiffusion Test for Quantification of Bovine IgG in Serum or Plasma, Kent Labs, Triple J Farms, Bellingham, WA, United States), as described by Dunn et al. (2018) and King et al. (2020). Plates were removed from refrigeration (4°C) and stored at room temperature (20°C) for approximately 1 h prior to the application of standards and test samples. Each sRID kit was supplied with three standard controls (low, 280 mg/dL; mid, 1,400 mg/dL; and high, 2,800 mg/dL), and 5 μL of each was applied to the plate. Serum samples were used neat or diluted appropriately (1:1 or 1:1.5) using 0.9% NaCl to fall within the range of the standard curve, and 5 μL of each applied to the plates. Plates were stored at room temperature (20°C–22°C) in the dark for 18 h. The diameters of the precipitin rings were measured using a digital caliper (0.150 mm digital caliper, Linear Tools 49-923-150, Linear Tools LTD, Middlesex, England, United Kingdom). The diameters of the rings were plotted against the IgG concentrations of the standards in Microsoft Excel to calculate the slope of the line, and the IgG concentrations of the samples (mg/ml) were calculated from the study by Dunn et al. (2018). The coefficient of determination (R^2^) values for the standard curves were between 0.95 and 0.98. The apparent efficiency of absorption (AEA) of IgG for each calf was calculated based on the calf’s weight, the volume of colostrum fed, and the IgG concentrations of both the colostrum and the calf’s blood serum using the same methodology as described by Conneely et al. (2014).

### 2.5 DNA extraction and sequencing

#### 2.5.1 Extraction and amplification

Sample processing order was determined on a random basis. Using a similar methodology as previously described (McGovern et al., 2018; Smith et al., 2020a), fecal microbial samples from the three sampling time-points (n = 150) were ground to fine powder, under liquid nitrogen, using a pestle and mortar. Microbial DNA was extracted from each sample using approximately 250 mg of ground feces and was subjected to repeated bead beating and column purification using the QIAGEN DNeasy® PowerSoil® Pro Kit (QIAGEN, Manchester, United Kingdom) and stored at −80°C. Microbial DNA extraction was also performed on the ZymoBIOMICS™ Microbial Community Standard (MC; Zymo Research Corp., Irvine, CA, United States), for each extraction kit (*n* = 9), as an internal positive control. DNA quality was assessed on 0.8% agarose gels with the concentration of extracted DNA quantified on the Nanodrop 1,000 spectrophotometer.

Extracted DNA was sent to Macrogen (Seoul, South Korea) for 16S rRNA amplicon library preparation and sequencing. In brief, the first round of PCR amplification, targeting the V4 hypervariable region of the 16S rRNA gene, was performed using the 515F/806R primers (Caporaso et al., 2011) designed with Nextera overhang adapters and Herculase II Fusion DNA Polymerase (Agilent, Santa Clara, CA, United States). Cycle conditions were as follows: 95°C for 3 min, 25 cycles at 95°C for 30 s, 55°C for 30 s, 72°C for 30 s, and then 72°C for 5 min. PCR amplicon purification was performed using the standard AMpure paramagnetic bead protocol (Beckman Coulter, Indianapolis, IN, United States). Following purification, amplicons were subjected to a second round of PCR to allow the attachment of dual indices and Illumina sequencing adapters using the Nextera XT indexing kit (Illumina, San Diego, CA, United States). Cycle conditions for the second round of PCR were as follows: 95°C for 3 min, 10 cycles at 95°C for 30 s, 55°C for 30 s, 72°C for 30 s, and then 72°C for 5 min followed by an additional PCR purification with the AMpure paramagnetic bead protocol (Beckman Coulter, Indianapolis, IN, United States). Amplicons were pooled together in equal concentration and were subjected to sequencing on the Illumina MiSeq using the 500 cycle version 2 MiSeq reagent kit (Illumina, San Diego, CA, United States) split equally across two flow cells.

#### 2.5.2 Sequencing analysis

Amplicon sequence data were processed in *R* (version 4.2.0) using *DADA2* (version 1.26.0) and submitted to the pipeline as described by Callahan et al. (2016), with minor modifications as outlined by Smith et al. (2020b, 2021). Quality check of both forward and reverse reads was carried out based on the visualization of Q scores, ensuring that the mean Q scores of >30 were upheld for forward and reverse reads. Filtering and trimming of poor quality reads and removal of primer sequences were conducted using the trimLeft function in *DADA2*. Identical sequences were combined using the de-replication function followed by the merging of forward and reverse reads. An amplicon sequence variant (ASV) table was then constructed following which chimeric sequences were removed and taxonomy was assigned to sequence variants using the SILVA database (version 138.1) downloaded from the *DADA2* website.^1^ A bootstrapping threshold of 80 was applied for taxonomic classification by incorporating minBoot = 80 as part of the assignTaxonomy function, as described by Smith et al. (2020b). Sample metadata, sequence taxonomy, and ASVs were combined into a phyloseq object using *phyloseq* (version 1.44.0; McMurdie and Holmes, 2013) and analyzed in *RStudio* (version 4.3.0).

**Figure 2:**
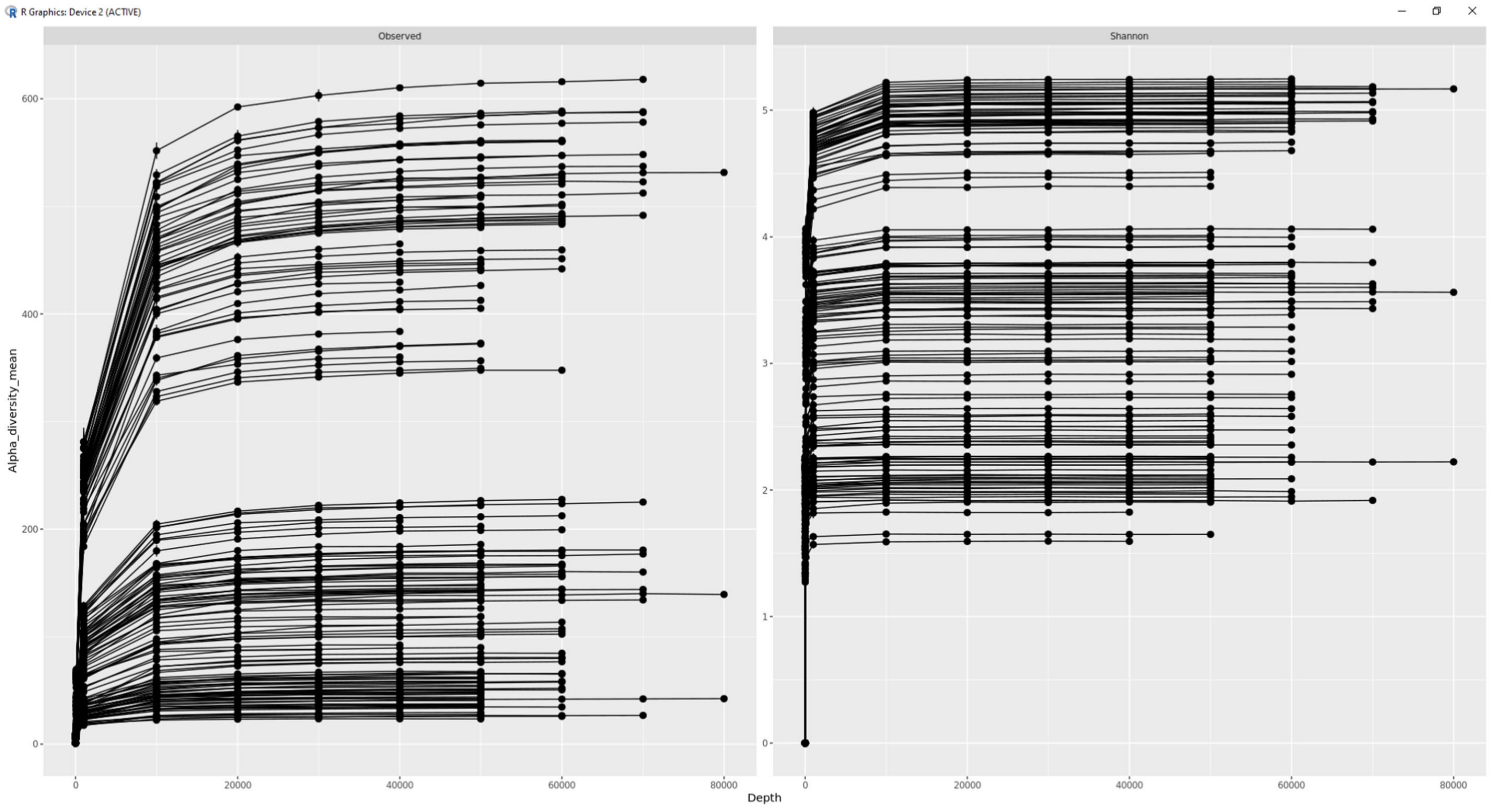
Rarefication curve illustrating sequencing depth.

A rarefication curve was plotted for fecal microbial samples (Figure 2). Based on plateauing of the generated rarefication curve, sequencing was deemed to be conducted to a sufficient depth. Following this, only bacterial ASVs, classified beyond the phylum level, were obtained. Subsequently, alpha (Shannon) diversity was calculated for each sample. For comparisons of beta diversity, as well as differential abundance analysis, ASVs which were not present in >0.05% of the samples were removed before calculating the relative abundance (RA).

### 2.6 Statistical analysis

#### 2.6.1 Calf performance and clinical assessment

Calf performance and clinical health datasets were analyzed using statistical procedures in SAS 9.4. Data for STP and IgG concentrations, average daily gain, and RT were checked for normality and homogeneity of variance by histograms, qqplots, and formal statistical tests as part of the UNIVARIATE procedure. Data were then analyzed using a repeated measures ANOVA (PROC MIXED procedure, SAS 9.4). The model included fixed effects (calf breed, dam breed, health status, and colostrum source) and random effects (shed and lactation number). Differences among means were determined by *F*-tests using Type III sums of squares. The PDIFF option and Tukey’s test were applied, where appropriate, to evaluate pairwise comparisons between means. Non-significant (*p* > 0.10) interactions were subsequently excluded from the final model. Mean values were considered statistically significant at *p* < 0.05, and tendency was considered when *p* > 0.05 and < 0.10. Pearson’s correlation coefficients were determined using the CORR procedure. Correlations (effect size and the strength of the correlation are denoted as r) were described as follows: 0.00–0.19 “very weak”; 0.20–0.39 “weak”; 0.40–0.59 “moderate”; 0.60–0.79 “strong”; 0.80–1.0 “very strong” (Swinscow, 1997). Correlations with *p* < 0.05 were considered statistically significant. Clinical health scores were analyzed using a Wilcoxon Rank Sum test (NPAR1WAY procedure).

#### 2.6.2 Sequencing data

The effect of health status and time-point on alpha diversity of the bacterial community was assessed using the PROC MIXED procedure in SAS. The model included fixed effects (calf breed, dam breed, health status, and colostrum source) and random effects (shed and lactation number). Differences among means were determined by *F*-tests using Type III sums of squares. The PDIFF option and results from Tukey’s test were applied to evaluate pairwise comparisons between means. Mean values were considered to be different when *p* < 0.05 and a tendency when *p* ≥ 0.05 and < 0.10.

Before assessing the effects of health status and time-point on the overall bacterial community structures, the homogeneity of group dispersion was assessed between groups. Following this, PERMANOVA tests based on Bray–Curtis dissimilarities, 9,999 permutations, and a significance level of p < 0.05 were implemented to determine if the bacterial community structure was impacted by calf health status, time-point, colostrum source, and calf breed. Both the assessment of the homogeneity of group dispersion and PERMANOVA analysis were carried out using the *R* package *vegan* (Oksanen et al., 2019; version 2.5.7) implemented through *microbiome* (Lahti and Shetty, 2017; version 1.12.0). Due to poor species level classification of ASVs, differential abundance analysis was performed at the genus, family, and phylum levels. Data were subset into time-points and then filtered for ASVs with an RA of >0.05% using *phyloseq*. At each time-point, statistical analysis of ASVs was performed using MaAsLin2 (version 1.14.1; Mallick et al., 2021) with time-point and health status as fixed effects and the Benjamini–Hochberg procedure to correct for false discovery rate. The three time-point files were then merged into one phyloseq object and run through the MaAsLin2 package with time-point and health status as fixed effect and the Benjamini–Hochberg procedure to correct for false discovery rate. The resulting data were used to determine the effect of time-point and health status on ASVs. While taxonomy was assigned using the SILVA database (version 138.1), phyla names were updated according to Oren and Garrity (2021). A Spearman rank-order correlation (r_s_) for non-parametric data was performed to determine correlations between ASVs and calf performance data and fecal scores at disease manifestation. Spearman correlations (effect and strength are denoted as r_s_) were described using the same methodology as outlined in section 2.6.1, and any correlations with *p* < 0.05 were considered significant.

## 3 Results

### 3.1 Clinical health

#### 3.1.1 Diarrheal disease incidence

Colostrum fed to calves had a mean Brix score of 26% (SE 0.51). Calves were weaned, on average, at d83 (SE 1.04; HO: d79, SE 1.22; JE: d88, SE 1.16) post-birth at an average weight of 82 kg (SE 1.10; HO: 85 kg, SE 1.38; JE: 79 kg, SE 1.50). Colostrum source and calf breed had no effect (*p* > 0.05) on disease incidence. Of the 51 calves enrolled, diarrheal incidence occurred in 53% (*n* = 27), with a mortality of 2% (*n* = 1) from birth to weaning. Day of diarrheal disease manifestation ranged from d18 to d38, with the mean day post-birth for diarrhea being d22 (SE 0.70).

#### 3.1.2 STP, IgG concentrations, and AEA

Serum IgG concentrations were strongly correlated (r = 0.61; *p* < 0.0001) with STP concentrations. There was no effect (*p* > 0.05) of colostrum source on calf STP, serum IgG concentrations, or AEA. Calf breed had no effect on mean STP or AEA (*p* > 0.05), whereas serum IgG concentrations were greater (*p* < 0.04) in Holstein calves than Jersey calves. Healthy calves and diarrheic calves had similar AEA, STP, and serum IgG concentrations (*p* > 0.05; Table 1).

**Table 1:** Passive immune status and calf performance.

	Breed (B)		Pooled SEM	Colostrum source (CS)		Pooled SEM	Health status (HS)		Pooled SEM	*p-*values^*^					
Passive immune variable	Holstein (*n* = 29)	Jersey (*n* = 22)		Dam (*n* = 28)	Mixed (*n* = 23)		Healthy (*n* = 24)	Diarrhea (*n* = 27)		B	CS	HS	B × CS	B × HS	CS × HS
Serum total protein (g/dL)	6.26	6.07	0.20	6.26	6.07	0.20	6.19	6.16	0.20	NS	NS	NS	NS	NS	NS
Serum IgG (mg/mL)	41.5	38.8	2.25	41.3	39.2	2.33	43.4	37.6	2.17	0.04	NS	NS	NS	NS	NS
Apparent Efficiency of Absorption (AEA)	24.03	22.69	1.54	23.26	23.67	1.61	25.25	21.70	1.51	NS	NS	NS	NS	NS	NS
Calf performance															
ADG (kg/d)	0.63	0.60	0.01	0.63	0.61	0.01	0.62	0.62	0.01	NS	NS	NS	NS	NS	NS

^*^p-value given where significant, NS, no significance (*P* > 0.05).

#### 3.1.3 Perinatal health scores

At d2 (SE 0.23) after birth, calves had a mean RT of 38.8°C (SE 0.07). At perinatal assessment, all calves were healthy. There was calf breed × colostrum source (*p* = 0.05) for RT where mixed colostrum Jersey calves tended to have a lower temperature than mixed colostrum HO (−0.40°C, SE 0.19; *p* = 0.05) and dam-fed JE calves (−0.40°C, SE 0.19; *p* < 0.05). There was no effect of calf breed or colostrum source on perinatal (baseline) health scores [data available on Open Science Framework (OSF)].

#### 3.1.4 Health scores pre-diarrheal disease—d7

At d7, calves had a mean RT of 39.1°C (SE 0.05). There were no calf breed × colostrum source interactions or effects of calf breed or colostrum source on RT prior to disease manifestation (DM; data available on OSF).

#### 3.1.5 Health scores disease manifestation—d21/DM

Calves were clinically diagnosed with diarrhea at a mean of d22 (SE 0.70) after birth. Health status had an effect on temperature (*p* < 0.0001), where healthy calves had lower (*p* = 0.0004) RT [38.9 (SE, 0.07)] than diarrheic calves [39.3 (SE 0.05)].

All health indicators were normal (median score of 0) with the exception of feces, which had a median score of 2 during disease manifestation (Table 2). There was no effect (*p* > 0.05) of colostrum source on any of the indicators that were used to assess health. Calf breed had an effect on joint scores (*p* < 0.0001), where HO had higher joint scores than JE. Health status had an effect on ear (*p* = 0.001), fecal (*p* < 0.0001), dehydration (*p* = 0.03), and mucus membrane (*p* = 0.05) scores such that diarrheic calves scored higher in these categories than healthy calves (Table 2).

**Table 2:** Clinical assessment at d21 (healthy calves) or disease manifestation (diarrheic calves).

	Breed (B)		Pooled SEM	Colostrum source (CS)		Pooled SEM	Health status (HS)		Pooled SEM	*P*-values^*^					
Variables	Holstein (*n* = 29)	Jersey (*n* = 22)		Dam (*n* = 28)	Mixed (*n* = 23)		Healthy (*n* = 24)	Diarrhea (*n* = 27)		B	CS	HS	B × CS	B × HS	CS × HS
Mean age at clinical assessment (d)	23	23	0.71	23	23	0.75	22	24	0.63	NS	NS	0.004	NS	NS	NS
Mean rectal body temperature (°C)	39.2	39.0	0.07	39.2	39.0	0.07	38.9	39.3	0.06	NS	NS	0.0004	NS	NS	NS
Health scores (median score)			Range			Range			Range						
Nasal	0	0	0–1	0	0	0–1	0	0	0–1	NS	NS	NS	NS	NS	NS
Ocular	0	0	0–1	0	0	0–1	0	0	0–1	NS	NS	NS	NS	NS	NS
Ear	0	0	0–2	0	0	0–2	0	0	0–2	NS	NS	0.001	NS	NS	NS
Cough	0	0	0–1	0	0	0–1	0	0	0–1	NS	NS	NS	NS	NS	NS
Feces	2	1	0–3	2	1	0–3	0	3	0–3	NS	NS	<0.0001	NS	NS	NS
Navel	0	0	0–2	0	0	0–2	0	0	0–2	NS	NS	NS	NS	NS	NS
Joints	1	0	0–2	0	0	0–2	0	1	0–2	<0.0001	NS	NS	NS	NS	NS
Coat	0	0	0–2	0	0	0–2	0	0	0–2	NS	NS	NS	NS	NS	NS
Dehydration	0	0	0–2	0	0	0–2	0	0	0–1	NS	NS	0.03	NS	NS	NS
Mucus membranes	0	0	0–1	0	0	0–1	0	0	0–1	NS	NS	0.05	NS	NS	NS

^*^NS, not statistically significant. Clinical assessment criteria are available on OSF.

#### 3.1.6 Health scores of post-diarrheal disease—d83

At d83 (SE 1.04) after birth, calves had a mean temperature of 39.0°C (SE 0.03). All indicators had a median score of 0 with the exception of ocular discharge, which had a median score of 1 after diarrheal disease. Colostrum source had an effect on calf temperature, where at weaning, dam-fed calves had a lower temperature (−0.17°C, SE 0.07; *p* = 0.01) than mixed colostrum calves. There were no other effects or interactions on calf temperature at weaning. Health status had an effect on navel score. There were no other interactions or effects on health scores at weaning (data available on OSF).

### 3.2 Performance data—ADG

There was no effect (*p* > 0.05) of colostrum source, calf breed, health status, or any interactions on ADG over the pre-weaning period (d0-d83). Calves were observed to perform with an ADG of 0.62 (SE 0.01) kg/d (Table 1). Healthy calves were weaned, on average, 4 days earlier (SE 1.74; *p* < 0.05) than diarrheic calves.

### 3.3 Fecal microbiota, temporal development, and health status

After quality filtering, merging, and removal of chimeric sequences, an average of 59,538 ± 9,854 reads per fecal sample were generated. Per time-point, fecal samples collected at pre-diarrheal disease (d7) produced an average of 56,294 ± 7,612 reads. Fecal samples collected at d21 or disease manifestation (d21/DM) generated an average of 60,200 ± 7,901 reads and those collected post-disease (d83) generated an average of 62,042 ± 8,192 reads. Correlations between positive controls ran for each kit were all highly correlated, with r_s_ ranging from 0.90 to 1.0 (*p* < 0.01).

#### 3.3.1 Characterization of the fecal microbiota during the pre-weaning period

Based on PERMANOVA analysis, calf breed (*p* = 0.08), colostrum source (*p* = 0.31), and passive immune status (*p* = 0.21) had no effect on the overall fecal microbiota composition. At the genus level, 396 unique ASVs were detected throughout the pre-weaning period across the three time-points (before diarrheal disease, during diarrheal disease, and after diarrheal disease). Genera and the adjusted *p*-values (adj.*p*) of ASVs associated with health status across the pre-weaning period are presented in Table 3. At the species level, 4,638 ASVs were detected. Over the pre-weaning period, four phyla dominated the fecal microbiota such as *Bacillota* (formerly known as *Firmicutes*—71%), *Bacteroidota* (15%)*, Actinomycetota* (formerly known as *Actinobacteria*—5%), and *Pseudomonadota* (formerly known as *Proteobacteria*—4%), which accounted for 95% of the RA of bacteria identified. Initially, before diarrheal disease, *Actinomycetota* (*Actinobacteria*—33%), *Pseudomonadota* (*Proteobacteria*—10%), and *Bacteroidota* (23%) made up the majority of microbiota present in the feces of all calves. As time progressed throughout the pre-weaning period, microbes within the *Bacillota* (*Firmicutes*) phylum began to proliferate, becoming the dominate phylum at disease manifestation (48%) and post-disease (52%) compared with pre-disease (34%).

**Table 3:** Genera and adjusted *p*-values (q) associated with health status during the pre-weaning period.

	Taxonomical classification		q-values^*^		
ASV	Phylum	Genus	Pre-disease transition^†^	During diarrhea^†^	Transition to recovery^†^
ASV1	*Actinomycetota (Actinobacteriota)*	*Bifidobacterium*	0.0003	0.0002	NS
ASV3	*Pseudomonadota (Proteobacteria)*	*Escherichia-Shigella*	0.04	0.04	0.02
ASV5	*Bacteroidota*	*Alloprevotella*	0.002	0.0002	0.0001
ASV6	*Actinomycetota (Actinobacteriota)*	*Collinsella*	NS	0.03	NS
ASV8	*Bacillota (Firmicutes)*	*Peptostreptococcus*	0.006	0.02	0.006
ASV9	*Bacteroidota*	*Prevotella*	NS	0.04	0.02
ASV12	*Fusobacteriiota*	*Fusobacterium*	0.04	NS	NS
ASV16	*Bacteroidota*	*Prevotella_9*	0.002	0.007	0.002
ASV18	*Bacillota (Firmicutes)*	*Ligilactobacillus*	0.0002	0.0009	0.0002
ASV20	*Bacillota (Firmicutes)*	*Rikenellaceae RC9 gut group*	0.04	NS	0.03
ASV22	*Bacillota (Firmicutes)*	*Phascolarctobacterium*	NS	0.02	0.03
ASV24	*Actinomycetota (Actinobacteriota)*	*Olsenella*	NS	NS	0.03
ASV25	*Bacteroidota*	NA	0.02	0.03	NS
ASV28	*Bacteroidota*	*Prevotella_7*	0.01	0.0009	0.0002
ASV41	*Bacteroidota*	*Prevotellaceae UCG-001*	NS	NS	0.03
ASV44	*Bacillota (Firmicutes)*	*Lachnospiraceae UCG-004*	0.02	0.03	NS
ASV48	*Bacillota (Firmicutes)*	*Faecalicoccus*	0.02	0.001	0.0004
ASV50	*Bacteroidota*	*Prevotellaceae UCG-003*	NS	NS	0.04
ASV58	*Bacillota (Firmicutes)*	NA	0.0004	0.001	0.0002
ASV66	*Bacillota (Firmicutes)*	NA	0.03	NS	NS
ASV67	*Bacillota (Firmicutes)*	*Erysipelatoclostridium*	NS	0.004	0.001
ASV75	*Thermodesulfobacteriota (Desulfobacterota)*	*Desulfovibrio*	0.02	0.03	0.02
ASV78	*Verrucomicrobioota*	*Akkermansia*	NS	0.04	0.03
ASV82	*Bacillota (Firmicutes)*	*Flavonifractor*	0.003	0.0003	0.0001
ASV88	*Bacillota (Firmicutes)*	NA	0.03	NS	0.03
ASV91	*Bacillota (Firmicutes)*	NA	0.002	0.007	0.0009
ASV92	*Bacillota (Firmicutes)*	*Allisonella*	0.006	0.02	0.007
ASV93	*Bacillota (Firmicutes)*	*Dialister*	0.006	0.02	0.006
ASV96	*Bacillota (Firmicutes)*	*Anaerofilum*	0.04	NS	0.05
ASV104	*Bacteroidota*	*Barnesiella*	NS	NS	0.03
ASV109	*Bacillota (Firmicutes)*	*Lachnospiraceae FCS020 group*	0.02	0.03	NS
ASV112	*Bacillota (Firmicutes)*	*Intestinibacter*	0.002	0.007	0.0008
ASV132	*Bacteroidota*	*Butyricimonas*	0.01	0.02	0.01

^*^ q = adjusted *p*-values determined using Benjamini–Hochberg procedure; NS, not statistically significant. ^†^Pre-disease transition, transition from pre-disease (d7) to disease manifestation; during diarrhea, at d21 (healthy calves) or disease manifestation (diarrheic calves); Transition to recovery, from disease manifestation to weaning (d83).

#### 3.3.2 Temporal establishment of the fecal microbiota

**Figure 3:**
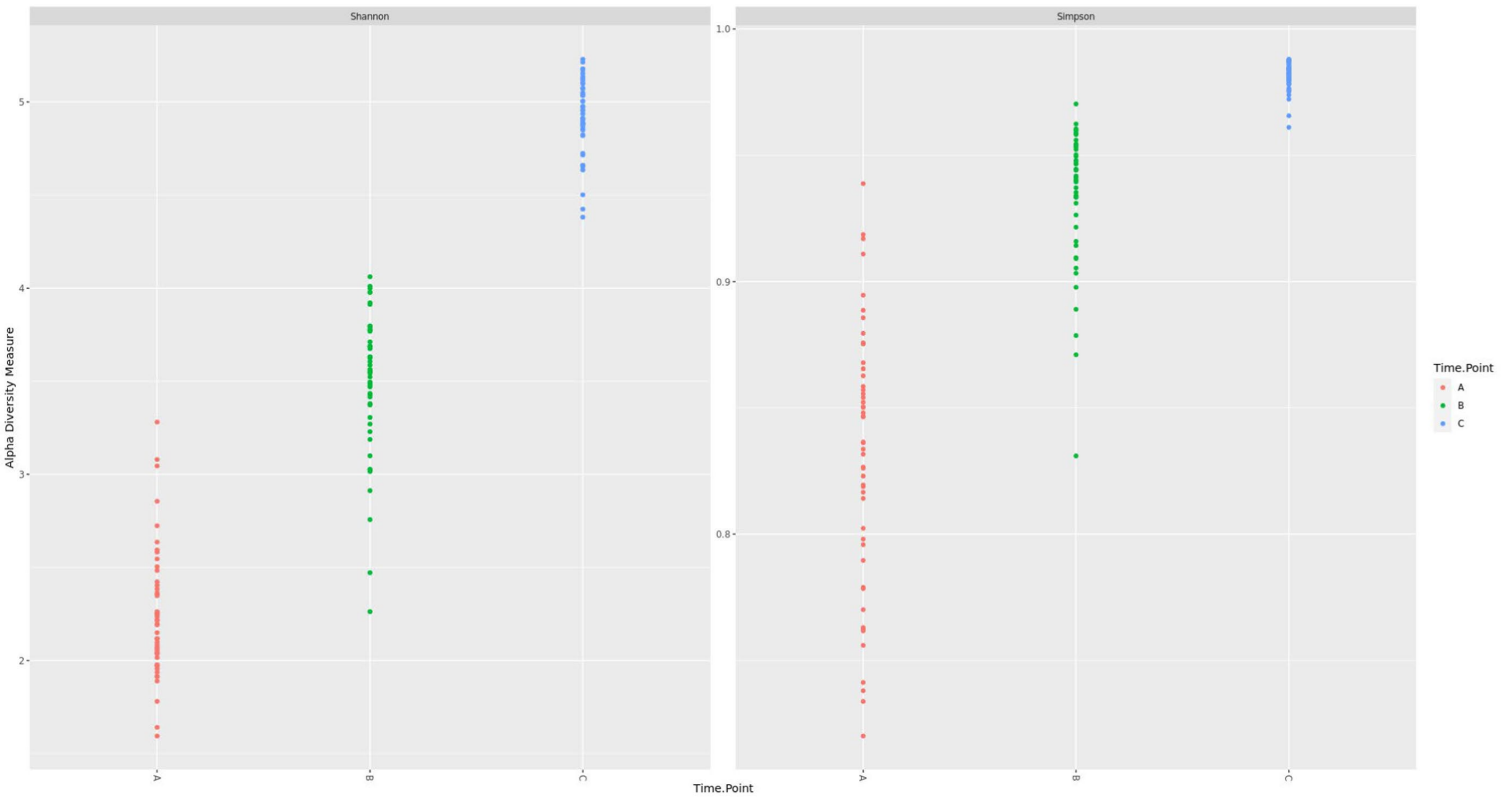
Shannon and Simpson diversity of the fecal microbiota during the pre-weaning period.

**Figure 4:**
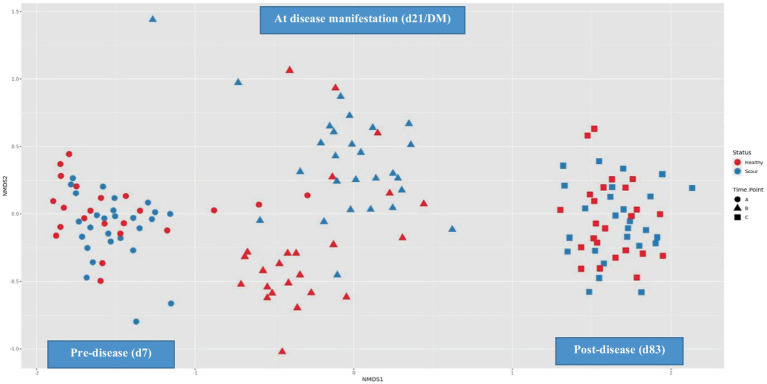
Temporal changes in beta diversity of the fecal microbiota from birth to weaning.

Before diarrheal disease, during disease manifestation, and after diarrheal disease, alpha diversity changed significantly (*p* = 0.0001) overtime. The fecal microbiota of calves became progressively more diverse during the pre-weaning period (Figure 3). Health status (*p* = 0.01) had a significant effect on the overall alpha diversity of the fecal microbiota. A significant interaction between time-point and health status (*p* = 0.0001) was observed, whereby at the second time-point (d21/DM), diarrheic calves had a reduction in microbial diversity (Figure 4). There was an effect of health status (*p* = 0.003) on differences in alpha diversity from d7 to d21/DM, and from d21/DM to d83, and none between d7 and d83.

#### 3.3.3 Effect of health status on the fecal microbiota

No ASVs were solely associated with health status on a genus level. At the species level, 9 ASVs were associated with health status during the pre-weaning period. Five of these ASVs were unclassified species within the *Prevotella_7* genus. The remaining four ASVs included *Faecolicoccus pleomorphus*, unclassified *Lachnoclostridium*, unclassified *Dialister,* and an unclassified microbe in the Ruminococcaceae family. *Lachnoclostridium* was the only ASV with a greater RA observed in diarrheic calves. All other ASVs were found to have a higher overall RA in healthy calves (Table 4).

**Table 4:** Species associated with health status and adjusted *p*-values (q) during the pre-weaning period.

ASV	Phylum	Genus	Species	RA-Diarrheic (%)	RA-Healthy (%)	q-value^*^
ASV52	*Bacteroidota*	*Prevotella_7*	NA	0.02	0.68	0.03
ASV128	*Bacillota (Firmicutes)*	*Lachnoclostridium*	NA	0.18	0.05	0.03
ASV167	*Bacteroidota*	*Prevotella_7*	NA	0.005	0.19	0.03
ASV251	*Bacteroidota*	*Prevotella_7*	NA	0.006	0.10	0.03
ASV152	*Bacteroidota*	*Prevotella_7*	NA	0.004	0.20	0.03
ASV172	*Bacillota (Firmicutes)*	*Faecalicoccus*	*pleomorphus*	0.04	0.14	0.03
ASV279	*Bacillota (Firmicutes)*	NA	NA	0.009	0.08	0.03
ASV224	*Bacillota (Firmicutes)*	*Dialister*	NA	0.02	0.10	0.04
ASV142	*Bacteroidota*	*Prevotella_7*	NA	0.006	0.22	0.04

RA Relative abundance (%). ^*^q value, adjusted *p*-value determined using Benjamini–Hochberg procedure.

##### 3.3.3.1 Pre-diarrheal disease—d7

At the pre-diarrheal disease time-point, all calves enrolled presented as healthy. No ASVs, from phyla to species level, were found to be significantly associated with health status at this time.

##### 3.3.3.2 Relationship of health status at d21/DM at the genus level

**Figure 5:**
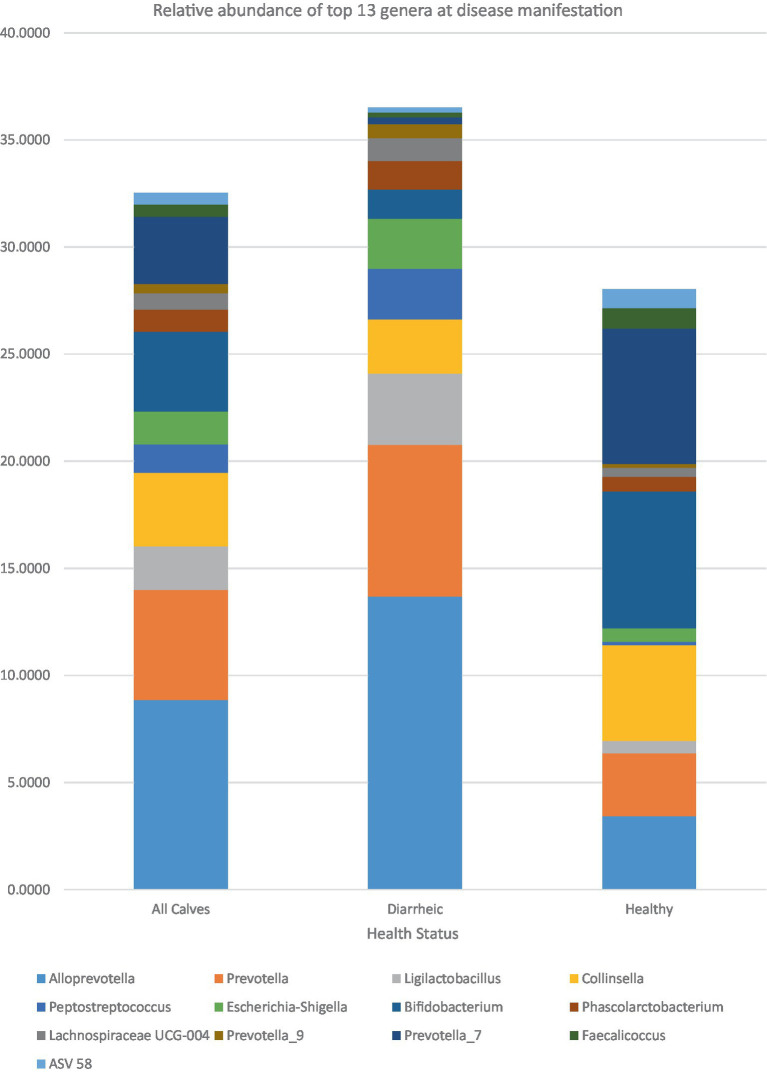
Relative abundance at disease manifestation (B).

At the genus level, 24 ASVs were significantly associated (adj.*p* < 0.05) with health status (Table 5). *Bifidobacterium, Flavonifractor, Prevotella_7, Faecalicoccus,* unclassified Ruminococcaceae, *Erysipelatoclostridium, Intestinibacter,* unclassified *Bacillota (Firmicutes)*, *Dialister, Butyricimonas, Collinsella,* Lachnospiraceae FCS020 group, *Desulfovibrio,* and *Akkermansia* were found to have a higher RA in healthy calves. *Alloprevotella, Ligilactobacillus, Prevotella_9, Allisonella, Phascolarctobacterium,* unclassified Prevotellaceae, Lachnospiraceae UCG-004, *Prevotella,* and *Escherichia-Shigella* were found to have a higher RA in diarrheic calves (Figure 5). At the species level, there were 43 ASVs identified at d21/DM, which were associated (adj.*p* < 0.05) with health status. Overall, 13 of those identified had a proportionally higher RA in diarrheic calves, and the remaining 30 were higher in healthy calves. *Bifidobacterium breve, Bifidobacterium pseudolongum,* and *Collinsella aerofaciens* and nine unclassified species of *Prevotella_7* were all proportionally higher in RA in the feces of healthy calves. In diarrheic calves, unclassified *Alloprevotella* was higher in RA than healthy calves. *Prevotella stercorea,* unclassified *Prevotella*, *Escherichia coli*, unclassified *Peptostreptococcus* (RA 2.36%, adj.*p* = 0.02), and 2 unclassified species of *Ligilactobacillus* (ASV 25: RA 2.87%, adj.*p* = 0.0004; ASV 154: RA 0.44%, adj.*p* = 0.01) were proportionally higher in diarrheic calves at disease manifestation.

**Table 5:** Relative abundance (%) of Genera associated with health status during disease manifestation (d21/DM).

ASV	Phylum	Genus	RA-Diarrheic	RA-Healthy	Difference	q-value^*^
ASV1	*Actinomycetota (Actinobacteriota)*	*Bifidobacterium*	1.36	6.38	−5.02	0.0002
ASV3	*Pseudomonadota (Proteobacteria)*	*Escherichia-Shigella*	2.33	0.63	1.70	0.04
ASV58	*Bacteroidota*	*Alloprevotella*	13.68	3.43	10.25	0.0002
ASV6	*Actinomycetota (Actinobacteriota)*	*Collinsella*	2.53	4.47	−1.94	0.03
ASV8	*Bacillota (Firmicutes)*	*Peptostreptococcus*	2.37	0.16	2.21	0.02
ASV9	*Bacteroidota*	*Prevotella*	7.08	2.94	4.15	0.04
ASV16	*Bacteroidota*	*Prevotella_9*	0.65	0.18	0.47	0.007
ASV18	*Bacillota (Firmicutes)*	*Ligilactobacillus*	3.33	0.58	2.75	0.0009
ASV22	*Bacillota (Firmicutes)*	*Phascolarctobacterium*	1.33	0.69	0.64	0.02
ASV 25	*Bacteroidota*	NA	0.53	0.30	0.23	0.03
ASV28	*Bacteroidota*	*Prevotella_7*	0.32	6.32	−6.00	0.0009
ASV44	*Bacillota (Firmicutes)*	*Lachnospiraceae UCG-004*	1.07	0.42	0.65	0.03
ASV48	*Bacillota (Firmicutes)*	*Faecalicoccus*	0.23	0.96	−0.73	0.001
ASV 58	*Bacillota (Firmicutes)*	NA	0.24	0.88	−0.64	0.001
ASV 67	*Bacillota (Firmicutes)*	*Erysipelatoclostridium*	0.16	0.48	−0.32	0.004
ASV 75	*Thermodesulfobacteriota (Desulfobacterota)*	*Desulfovibrio*	0.11	0.39	−0.29	0.03
ASV78	*Verrucomicrobiota*	*Akkermansia*	0.10	0.25	−0.15	0.04
ASV82	*Bacillota (Firmicutes)*	*Flavonifractor*	0.09	0.45	−0.36	0.0003
ASV 91	*Bacillota (Firmicutes)*	NA	0.09	0.29	−0.21	0.007
ASV92	*Bacillota (Firmicutes)*	*Allisonella*	0.26	0.06	0.20	0.02
ASV93	*Bacillota (Firmicutes)*	*Dialister*	0.07	0.41	−0.34	0.02
ASV109	*Bacillota (Firmicutes)*	*Lachnospiraceae FCS020 group*	0.08	0.14	−0.07	0.03
ASV112	*Bacillota (Firmicutes)*	*Intestinibacter*	0.05	0.21	−0.16	0.007
ASV132	*Bacteroidota*	*Butyricimonas*	0.04	0.2	−0.16	0.02

RA relative abundance (%), difference (diarrheic % − healthy %) expressed as percentage points. ^*^q-value, adjusted *p*-value determined using Benjamini–Hochberg procedure.

##### 3.3.3.3 Post-diarrheal disease—d83

At d83 (post-diarrheal disease), all calves presented as healthy, and no ASVs, from phyla to species level, were associated with health status.

#### 3.3.4 Comparison of the progression of the fecal microbiota between healthy and diarrheic calves

##### 3.3.4.1 Transition from pre-disease to disease manifestation

At pre-disease, no ASVs were associated with health status; however, when analyzing the changes in the RA of ASVs from pre-disease to disease manifestation, 25 genera were significantly (adj.*p* < 0.05) associated with health status during this transition period. In both healthy and diarrheic calves, there was a decrease in the RA of *Bifidobacterium* spp. (adj.*p* < 0.001) from pre-disease to disease manifestation, with a larger loss observed in diarrheic calves. There was a decrease in RA of *Escherichia-Shigella* (adj.*p* < 0.05) in both cohorts; however, this decrease was greater in healthy calves. The RA of *Alloprevotella* (adj.*p* < 0.01) increased in both cohorts; however, the increase was proportionally greater in diarrheic calves, as opposed to healthy calves. Similar to *Alloprevotella*, an increase was observed in both cohorts in the RA of *Ligilactobacillus* (adj.*p* < 0.001), but a greater increase was observed in diarrheic calves. *Fusobacterium* (adj.*p* < 0.05) followed a similar trend such that the RA increased from pre-disease to disease manifestation for both cohorts, but diarrheic calves experienced a greater increase. With regard to *Peptostreptococcus* (adj.*p* < 0.01), a decrease was observed in RA in both healthy and diarrheic calves; however, the RA pre-disease in diarrheic calves was higher (RA 5.8%) than in healthy calves (RA 3.4%). The RA of *Prevotella_7* (adj.*p* = 0.01) decreased from 0.53% to 0.31% in diarrheic calves and increased from 0.02% to 6.32% in healthy calves (data available on OSF).

##### 3.3.4.2 Transition from disease manifestation to post-disease

When analyzing the changes in the RA of ASVs from disease manifestation to post-disease, 26 genera or 53 species were significantly (adj.*p* < 0.05) associated with calf health status. When moving from disease manifestation to post-disease, at weaning, at the genus level, *Bifidobacterium* was no longer significantly associated with health status; however, at the species level, *Bifidobacterium breve* was significantly (adj.*p* < 0.001) associated with health status. In case of *Bifidobacterium breve,* a larger decrease was observed in the RA of healthy calves. In case of both cohorts, a decrease was observed in RA of *Escherichia-Shigella* (adj.*p* < 0.05) through the transition from disease manifestation to post-disease. During the transition from disease to post-disease, diarrheic calves showed a decrease in the RA of *Alloprevotella* (adj.*p* = 0.0001), while healthy calves showed an increase. *Peptostreptococcus* (adj.*p* < 0.01) and *Ligilactobacillus* (adj.*p* < 0.001) decreased in both groups. Similarly, *Prevotella_7* (adj.*p* < 0.001) decreased in RA in healthy calves and diarrheic calves. There was a greater increase in RA of *Prevotella* (adj.*p* < 0.05) in healthy calves between these time-points (data available on OSF).

### 3.4 Correlations

#### 3.4.1 Calf performance, immune status, and ASVs

Serum total protein and serum IgG concentrations were moderately and weakly correlated with a variety of ASVs associated with health status in all calves, diarrheic calves, and healthy calves. Moderate correlations were observed between ASVs and ADG in diarrheic calves and in healthy calves (Table 6). Across all calves at disease manifestation, correlations between fecal score and all ASVs significantly associated with health status ranged from weak to strong (Table 6). There was a strong positive correlation between fecal score at disease manifestation and *Alloprevotella* (r_s_ = 0.61, *p* ≤ 0.0001). Fecal scores were observed to have a moderate negative correlation with *Bifidobacterium*, *Colinsella*, *Prevotella_7*, *Faecalicoccus*, ASV 58, *Erysipelatoclostridium*, *Akkermansia*, *Flavonifractor*, ASV 91, *Dialister*, and *Intestinibacter*. Moderate positive correlations were observed between fecal scores and *Prevotella_9*, *Ligilactobacillus*, ASV 25, and *Allisonella*.

**Table 6:** Spearman rank-test correlation coefficients (r_s_) for ASVs and calf performance, immune status, and fecal scores.

	Average daily gain			Serum total protein			Serum IgG concentration			Fecal score		
Genus	All	Diarrheic	Healthy	All	Diarrheic	Healthy	All	Diarrheic	Healthy	All	Diarrheic	Healthy
*Bifidobacterium*	NS	NS	NS	NS	NS	NS	NS	NS	NS	−0.58^***^	NS	NS
*Escherichia-Shigella*	NS	NS	NS	NS	NS	NS	NS	NS	NS	0.31	NS	NS
*Alloprevotella*	NS	NS	NS	NS	NS	NS	−0.35^*^	NS	NS	0.61^***^	NS	NS
*Collinsella*	NS	NS	NS	NS	NS	NS	NS	NS	NS	−0.43^*^	NS	NS
*Peptostreptococcus*	NS	NS	−0.47	−0.45^**^	−0.42	−0.57^*^	−0.35^*^	−0.43	NS	0.39^*^	NS	NS
*Prevotella*	NS	NS	NS	−0.37^**^	NS	−0.46	−0.37^*^	−0.38	NS	0.33	NS	NS
*Prevotella_9*	NS	NS	NS	NS	NS	NS	−0.27	NS	NS	0.52^***^	NS	NS
*Ligilactobacillus*	NS	NS	NS	NS	NS	NS	−0.28	NS	NS	0.56^***^	NS	NS
*Phascolarctobacterium*	NS	NS	NS	NS	NS	NS	NS	NS	NS	0.38^*^	NS	NS
ASV 25 (Family-*Prevotellaceae*)	NS	NS	NS	NS	NS	NS	NS	NS	NS	0.42^*^	NS	NS
*Prevotella_7*	NS	NS	NS	0.37^*^	0.43	0.47	0.46^**^	0.51^*^	NS	−0.57^***^	NS	NS
*Lachnospiraceae UCG-004*	NS	NS	NS	NS	NS	NS	NS	NS	0.40	0.36^*^	NS	NS
*Faecalicoccus*	NS	NS	NS	0.31	0.45	NS	0.40^*^	0.49^*^	NS	−0.54^***^	NS	NS
ASV 58 (Family *Ruminococcaceae*)	NS	NS	0.42	0.27	NS	NS	0.35^*^	0.44	NS	−0.58^***^	NS	NS
*Erysipelatoclostridium*	NS	NS	NS	NS	NS	0.42	NS	NS	NS	−0.45^**^	NS	NS
*Desulfovibrio*	NS	0.55^*^	NS	NS	NS	NS	0.32	0.43	NS	−0.34	NS	NS
*Akkermansia*	NS	NS	NS	NS	NS	NS	0.30	0.43	NS	−0.41^*^	NS	NS
*Flavonifractor*	NS	NS	NS	0.31	NS	NS	NS	NS	NS	−0.54^***^	NS	NS
ASV 91 (Phylum: Bacillota)	NS	NS	NS	0.37^*^	NS	0.59^*^	0.33	0.40	NS	−0.46^**^	NS	NS
*Allisonella*	NS	NS	NS	NS	NS	NS	NS	NS	NS	0.50^**^	NS	NS
*Dialister*	NS	NS	NS	NS	NS	NS	NS	NS	NS	−0.40^*^	NS	NS
*Lachnospiraceae FCS020 group*	NS	NS	0.53^*^	0.38^*^	NS	0.49°	NS	NS	NS	0.38^*^	NS	NS
*Intestinibacter*	NS	NS	NS	NS	NS	NS	NS	NS	NS	−0.50^**^	NS	NS
*Butyricimonas*	NS	NS	NS	NS	NS	NS	0.30	NS	NS	−0.37^*^	NS	NS

NS; Not statistically significant (*P* > 0.05); No annotation-*p* ≤ 0.05. ^*^p ≤ 0.01. ^**^p ≤ 0.001. ^***^p ≤ 0.0001.

#### 3.4.2 Correlations between ASVs at disease manifestation

At the time of disease manifestation, 29 strong correlations (r_s_ range as absolute value: 0.60–0.77), 72 moderate correlations (r_s_ range as absolute value: 0.40–0.59), and 72 weak correlations (r_s_ range as absolute value: 0.27–0.39) of significance (*p* ≤ 0.05) were observed between the various genera associated with the health status (Table 7).

**Table 7:** Spearman rank-test correlation coefficients (r_s_) inter-ASVs.

Genus	*Bifidobacterium*	*Escherichia-Shigella*	*Alloprevotella*	*Collinsella*	*Peptostreptococcus*	*Prevotella*	*Prevotella_9*	*Ligilactobacillus*	*Phascolarctobacterium*	*ASV 25*	*Prevotella_7*	*Lachnospiraceae UCG-004*	*Faecalicoccus*	*ASV 58*	*Erysipelatoclostridium*	*Desulfovibrio*	*Akkermansia*	*Flavonifractor*	*ASV 91*	*Allisonella*	*Dialister*	*Lachnospiraceae FCS020 group*	*Intestinibacter*	*Butyricimonas*
*Bifidobacterium*	NS	−0.35^*^	−0.50^**^	0.58^***^	−0.45^**^	−0.47^**^	NS	−0.53^***^	−0.28	−0.36^*^	0.62^***^	NS	0.56^***^	0.39^*^	0.31	NS	0.33	0.52^***^	NS	NS	0.52^***^	0.32	0.42^*^	NS
*Escherichia-Shigella*	−0.35^*^	NS	NS	−0.31	0.52^***^	NS	NS	NS	NS	NS	−0.49^**^	NS	−0.41^*^	−0.38^*^	−0.30	NS	NS	−0.43^**^	−0.39^*^	NS	−0.32	−0.44^**^	NS	NS
*Alloprevotella*	−0.50^**^	NS	NS	NS	0.43^*^	0.57^***^	0.64^***^	0.58^***^	0.63^***^	0.71^***^	−0.58^***^	0.36^*^	−0.60^***^	−0.58^***^	−0.49^**^	−0.28	−0.68^***^	−0.45^**^	−0.39^*^	0.35^*^	−0.40^*^	NS	−0.61^***^	−0.35^*^
*Collinsella*	0.58^***^	−0.31	NS	NS	−0.29	NS	NS	−0.41^*^	NS	NS	0.40^*^	NS	0.29	0.33	NS	NS	NS	0.39^*^	NS	NS	NS	NS	NS	NS
*Peptostreptococcus*	−0.45^**^	0.52^***^	0.43^*^	−0.29	NS	0.43^**^	NS	0.42^*^	NS	NS	−0.61^***^	NS	−0.52^***^	−0.67^***^	−0.30	NS	−0.43^*^	−0.63^***^	−0.66^***^	0.46^**^	−0.33	−0.70^***^	−0.37^*^	−0.34^*^
*Prevotella*	−0.47^**^	NS	0.57^***^	NS	0.43^**^	NS	0.34^*^	0.36^*^	0.36^*^	0.51^***^	−0.62^***^	NS	−0.59^***^	−0.39^*^	−0.28	NS	−0.50^**^	NS	−0.27	NS	−0.40^*^	−0.27	−0.64^***^	NS
*Prevotella_9*	NS	NS	0.64^***^	NS	NS	0.34^*^	NS	0.77^***^	0.45^**^	0.74^***^	−0.39^*^	NS	−0.55^***^	−0.41^*^	−0.35^*^	NS	NS	NS	NS	NS	NS	NS	−0.29	NS
*Ligilactobacillus*	−0.53^***^	NS	0.58^***^	−0.41^*^	0.42^*^	0.36^*^	0.77^***^	NS	0.49^***^	0.61^***^	−0.54^***^	NS	−0.61^***^	−0.46^**^	−0.36^*^	NS	−0.50^**^	−0.30	NS	0.30	−0.42^*^	NS	−0.37^*^	NS
*Phascolarctobacterium*	−0.28	NS	0.63^***^	NS	NS	0.36^*^	0.45^**^	0.49^***^	NS	0.62^***^	−0.33	0.39^*^	NS	NS	NS	NS	−0.42^*^	NS	NS	NS	−0.44^**^	NS	−0.38^*^	NS
*ASV 25*	−0.36^*^	NS	0.71^***^	NS	NS	0.51^***^	0.74^***^	0.61^***^	0.62^***^	NS	−0.40^*^	NS	−0.59^***^	−0.34^*^	−0.37^*^	NS	−0.64^***^	NS	NS	NS	−0.29	NS	−0.39^*^	NS
*Prevotella_7*	0.62^***^	−0.49^**^	−0.58^***^	0.40^*^	−0.61^***^	−0.62^***^	−0.39^*^	−0.54^***^	−0.33	−0.40^*^	NS	NS	0.69^***^	0.61^***^	0.41^*^	NS	0.44^**^	0.56^***^	0.57^***^	−0.29	0.70^***^	0.55^***^	0.33	NS
*Lachnospiraceae UCG-004*	NS	NS	0.36^*^	NS	NS	NS	NS	NS	0.39^*^	NS	NS	NS	NS	NS	NS	NS	NS	NS	NS	NS	0.32	NS	NS	NS
*Faecalicoccus*	0.56^***^	−0.41^*^	−0.60^***^	0.29	−0.52^***^	−0.59^***^	−0.55^***^	−0.61^***^	NS	−0.59^***^	0.69^***^	NS	NS	0.62^***^	0.42^*^	0.41^*^	0.62^***^	0.50^**^	0.29	−0.33	0.48^**^	0.42^*^	0.49^**^	0.47^**^
*ASV 58*	0.39^*^	−0.38^*^	−0.58^***^	0.33	−0.67^***^	−0.39^*^	−0.41^*^	−0.46^**^	NS	−0.34^*^	0.61^***^	NS	0.62^***^	NS	0.29	0.32	0.52^***^	0.52^***^	0.50^**^	−0.52^***^	0.37^*^	0.55^***^	0.39^*^	0.49^**^
*Erysipelatoclostridium*	0.31	−0.30	−0.49^**^	NS	−0.30	−0.28	−0.35^*^	−0.36^*^	NS	−0.37^*^	0.41^*^	NS	0.42^*^	0.29	NS	0.35^*^	0.36^*^	0.43^**^	NS	NS	0.29	0.38^*^	0.51^***^	0.31
*Desulfovibrio*	NS	NS	−0.28	NS	NS	NS	NS	NS	NS	NS	NS	NS	0.41^*^	0.32	0.35^*^	NS	0.41^*^	NS	NS	NS	NS	NS	NS	0.75^***^
*Akkermansia*	0.33	NS	−0.68^***^	NS	−0.43^*^	−0.50^**^	NS	−0.50^**^	−0.42^*^	−0.64^***^	0.44^**^	NS	0.62^***^	0.52^***^	0.36^*^	0.41^*^	NS	NS	NS	NS	0.36^*^	NS	0.48^**^	0.41^*^
*Flavonifractor*	0.52^***^	−0.43^**^	−0.45^**^	0.39^*^	−0.63^***^	NS	NS	−0.30	NS	NS	0.56^***^	NS	0.50^**^	0.52^***^	0.43^**^	NS	NS	NS	0.60^***^	−0.51^***^	0.31	0.71^***^	0.41^*^	0.39^*^
*ASV 91*	NS	−0.39^*^	−0.39^*^	NS	−0.66^***^	−0.27	NS	NS	NS	NS	0.57^***^	NS	0.29	0.50^**^	NS	NS	NS	0.60^***^	NS	−0.37^*^	0.33	0.74^***^	0.30	NS
*Allisonella*	NS	NS	0.35^*^	NS	0.46^**^	NS	NS	0.30	NS	NS	−0.29	0.32	−0.33	−0.52^***^	NS	NS	NS	−0.51^***^	−0.37^*^	NS	NS	−0.43^**^	−0.38^*^	−0.31
*Dialister*	0.52^***^	−0.32	−0.40^*^	NS	−0.33	−0.40^*^	NS	−0.42^*^	−0.44^**^	−0.29	0.70^***^	NS	0.48^**^	0.37^*^	0.29	NS	0.36^*^	0.31	0.33	NS	NS	0.32	0.39^*^	NS
*Lachnospiraceae FCS020 group*	0.32	−0.44^**^	NS	NS	−0.70^***^	−0.27	NS	NS	NS	NS	0.55^***^	NS	0.42^*^	0.55^***^	0.38^*^	NS	NS	0.71^***^	0.74^***^	−0.43^**^	0.32	NS	NS	NS
*Intestinibacter*	0.42^*^	NS	−0.61^***^	NS	−0.37^*^	−0.64^***^	−0.29	−0.37^*^	−0.38^*^	−0.39^*^	0.33	NS	0.49^**^	0.39^*^	0.51^***^	NS	0.48^**^	0.41^*^	0.30	−0.38^*^	0.39^*^	NS	NS	0.28
*Butyricimonas*	NS	NS	−0.35^*^	NS	−0.34^*^	NS	NS	NS	NS	NS	NS	NS	0.47^**^	0.49^**^	0.31	0.75^***^	0.41^*^	0.39^*^	NS	−0.31	NS	NS	0.28	NS

NS, Not statistically significant (*P* > 0.05); No annotation, *P* ≤ 0.05. ^*^P ≤ 0.01. ^**^P ≤ 0.001. ^***^P ≤ 0.0001.

## 4 Discussion

The present study examined the effect of health status on the development of the fecal microbiota of Jersey and Holstein heifer calves during the pre-weaning period. There was little variation in serum IgG concentrations between healthy and diarrheic calves, indicating that diarrheal disease manifested despite adequate immune status. All calves were determined to be healthy, with no difference in fecal microbiota composition before disease. The same was observed after disease, indicating that all calves started similarly and those that did develop diarrhea were able to adequately recover and re-establish an equilibrium within the hindgut microbiota. The results of the present study confirm dysbiosis of the hindgut microbiome, resulting in diarrheal disease, and provide an insight into the changes in microbial composition that may lead to dysbiosis, which enables diarrheal disease manifestation.

### 4.1 Calf management and disease incidence

Calf breed and colostrum source had no effect on diarrheal incidence. Additionally, there was little variation in serum IgG concentrations between cohorts (diarrheic, healthy), indicating that diarrheal incidence is due to other factors and manifested independently of transfer of passive immunity. The strong correlation between serum IgG and STP indicate that STP is an adequate proxy for evaluating serum IgG as previously reported by Wilm et al. (2018).

In the current study, there was no effect of colostrum source on serum IgG, such that serum IgG was similar for calves that received colostrum from their own dam and a mixed source. The present findings are in agreement with those reported by King et al. (2020).

In the current study, diarrheal disease manifestation could be attributed to dysbiosis of the hindgut microbiome (Gomez et al., 2017), environmental pathogen load, changing diet, or some other stressors, all of which impact immune function (Hulbert and Moisá, 2016) and microbiome development (Amin and Seifert, 2021; Du et al., 2023). In addition, calf health status had no effect on the average daily gain from birth to weaning, indicating that calves performed the same, regardless of diarrheal disease manifestation. Sayers et al. (2016) reported that treating neonatal calf diarrhea with oral rehydration solution (electrolytes), as were the calves in the current study, enhances calf recovery, suggesting that the appropriate use of supportive therapies enable diarrheic calves to recover without an effect on performance.

### 4.2 Effects on the fecal microbiota

#### 4.2.1 Temporal development

In the current study, the temporal development of the fecal microbiota observed during the pre-weaning period is in agreement with the findings reported by Song et al. (2018), Hennessy et al. (2020), and Massot et al. (2020), such that overall diversity of the fecal microbiota increased with time, supporting the assertion that microbial colonization of the lower gastrointestinal tract of calves occurs sequentially and progressing rapidly from birth to weaning as has been reported by Alipour et al. (2018).

#### 4.2.2 Colostrum management

Neonatal calves are pseudo-monogastric, relying on a milk-based diet, with the main site of digestion, via microbial fermentation, occurring in the small and large intestines (Amin and Seifert, 2021). Inoculation of the hindgut comes from a variety of sources including the placenta and other maternal factors, as well as initial diet and feeding regime (Amin and Seifert, 2021; Du et al., 2023). Milk oligosaccharides are a critical energy source for the initial microbial community (Boudry et al., 2017). The feeding of colostrum has been reported to influence the initital colonization of the hindgut, in that calves that were fed colostrum (fresh and heat-treated) had an increased presence of *Bifidobacterium* in the first 12 h of life, and feeding heat-treated colostrum may be even more beneficial, even though *Bifidobacterium* was not present in the colostrum fed (Song et al., 2019). Calves are relied on high-quality colostrum for successful passive transfer of immunity from the dam (McGee and Earley, 2019). The combination of immunoglobulins, antimicrobial factors, growth factors, anti-inflammatories, and nutrients in colostrum are essential for calf health and development (Carter et al., 2021). The nutrients and other bio-active compounds, e.g., lactoferrin, present in colostrum are important for pioneering bacteria that are colonizing the hindgut (Fischer-Tlustos et al., 2021). The feeding of colostrum helps to inhibit pathogen growth, stimulate colonization of the small intestine with commensal microbes, increased weight gain, improved development and function of the gastrointestinal tract, and reduce the risk of diarrhea (Song et al., 2019; Amin and Seifert, 2021; Carter et al., 2021; Fischer-Tlustos et al., 2021). It has been suggested that bacteria found in colostrum may influence the colonization and proliferation of early life microbial communities in the calf hindgut (Gomez et al., 2019). In the current study, calves were handfed colostrum collected using different management practices, colostrum that was collected directly from the calf’s own dam and fed to the calf or colostrum that was sourced from one to two donors, refrigerated and re-heated. Colostrum from a refrigerated, mixed source is at higher risk of environmental contamination (Godden et al., 2019). Increased environmental contamination with microbes could possibly lead to increased risk of disease (Godden et al., 2019) but also increased number of microbes inoculating the hindgut. Thus, one could hypothesize that these different management practices may have had an effect on disease incidence and the composition of the fecal microbiota of the calf. However, the different colostrum sources had neither an effect on diarrheal disease manifestation nor an effect on the diversity or composition of the fecal microbiota at any of the sampling time-points during the pre-weaning period. This may be related to multiple factors in the animal model of the current study. First, if fed quality colostrum, the source of the colostrum (own dam, different dam, pooled) has no effect on serum IgG and passive transfer of immunity, as was reported by King et al. (2020). There was little variation in immune status between diarrheic and healthy calves, and any effect of colostrum source on serum IgG, implying that any changes observed in the fecal microbiota, is neither associated with diarrheal disease manifestation nor poor passive transfer of immunity. Second, the mixed colostrum that was stored in a refrigerator, was collected from the dam and stored as quickly as possible, had been refrigerated for no more than 24 h and was heat-treated, according to best practices prior to feeding. These practices are in line with best practices for minimizing bacterial contamination as suggested by Godden et al. (2019). Additionally, as has been previously discussed, Song et al. (2019) have reported that the feeding of heat-treated colostrum (60°C for 60 min) increases the abundance of beneficial genera such as *Bifidobacterium*. Lastly, from second feed to d14 all calves were fed re-heated, pooled-transition milk sourced from the maternal herd.

#### 4.2.3 Calf breed

Host genetics has previously been suggested to influence the composition of the hindgut microbiota (Paz et al., 2016; Fan et al., 2020; Slanzon et al., 2022). However, in contrast to the findings by Slanzon et al. (2022), there was no effect of calf breed on the diversity and overall composition of the fecal microbiota in the current study. The calves enrolled in the study by Slanzon et al. (2022) were of similar breeds to those of the present study (Holstein, Jersey, and Jersey-cross) with the exception of beef-crosses and were 4–21 days of age. Differences between the present study and others may be due to a variety of factors, such as multiple sampling time-points vs. singular ones, calf age and life stage, or calf-farm origin and other environmental factors. The difference in the findings between the present study and the study by Slanzon et al. (2022) could be due to calf origin and different diets. Slanzon et al. (2022) found that breed had an effect on the fecal microbiome of calves, all of which originated on different farms and were then reared indoors together. The differences, however, were most evident in calves with or without gastrointestinal disease rather than between breeds (Slanzon et al., 2022). The calves enrolled in the study by Slanzon et al. (2022) were administered two different milk diets depending on enrollment date, which included pasteurized waste milk, whereas calves in the present study were home bred and fed colostrum artificially from within the maternal herd. Additionally, calves were fed transition milk sourced from the maternal herd until d14 when they were transitioned to milk replacer. This difference in management may suggest that breed differences detected in early life may in fact be related to differences in environment and feeding strategies. The lack of effect of breed on diversity and composition in the present study may also be due to age. All calves were enrolled, and all sampling time-points occurred during the pre-weaning period. Fan et al. (2020) found that host genetics did have an effect on composition in animals at 3 months of age, while no differences were detected in calves at a similar age (d83) in the present study. However, Fan et al. (2020) examined the effect of species and crossbreeding on the early gut microbiota of calves ranging from 100% Angus to 100% Brahman, with varying degrees of cross-breeding between the two mid-points. While there were two different breeds (Holstein × and Jersey ×) enrolled in the present study, they were of the *Bos Taurus* species, suggesting that at the species level, host-genetics may have an effect on the composition of the fecal microbiota. At maturity, an effect of breed has been observed in the composition of the rumen bacterial community, such that the composition of the rumen microbiota varied between lactating Holstein and Jersey cows (Paz et al., 2016) reflecting the findings reported by Fan et al. (2020), regarding the relationship between age, host-genetics, and composition. However, even with a period of acclimation, the Jersey cows sampled by Paz et al. (2016) were from a different farm origin than the Holstein cows that were used for comparison. The lack of breed effect on composition and diversity in the present study may be related to age, although no breed effect was observed at post-disease, where calves were approximately 3 months old. The findings of the current study, however, suggest that in the aforementioned studies, an effect of farm origin may be coming as an apparent effect of breed and host genetics.

### 4.3 The fecal microbiota at disease manifestation

Similar to other studies (Gomez et al., 2017, 2022; Slanzon et al., 2022), at disease manifestation, a reduction in the diversity of the bacterial community in diarrheic calves was observed. Of the 24 genera found to be significant at disease manifestation, *Bifidobacterium* spp. was significantly associated with health status, with a higher relative abundance in healthy calves, which was similar to previous findings (Gomez et al., 2017; Slanzon et al., 2022). However, the species identified, differed in the study by Slanzon et al. (2022), who found *Bifidobacterium longum* to be associated with gastrointestinal health, while in the present study, *B. breve* and *B. pseudolongum* were present. This difference could be due to environmental factors associated with the calf rearing environment, as it has previously been reported that farm environment influences the composition of microbial communities (Gomez et al., 2017; O'Hara et al., 2020).

The role of *Bifidobacterium* in the hindgut, regardless of species, remains the same. *Bifidobacterium* has been recognized for its probiotic effect, since Abe et al. (1995) associated it with decreased diarrheal disease. This genus, a known “milk-user,” appears in high relative abundance in the hindgut of neonatal calves (Gomez et al., 2019). The oligosaccharides present in bovine milk promote the proliferation of this genus (Boudry et al., 2017) when milk consumption supersedes solid feed intake. *Bifidobacterium* promotes the production of butyrate by producing acetate which is then used by butyrate-producing bacteria (Rivière et al., 2016). Butyrate is critical in the development and maintenance of the lower GIT, the epithelial lining, and mucosal immune response and modulation (Wang et al., 2012; Arpaia et al., 2013; Gomez et al., 2019; Fischer-Tlustos et al., 2021). *Bifidobacterium* has also been observed to inhibit the growth and proliferation of enteric pathogens by outcompeting them for epithelial binding sites (Picard et al., 2005; Aw and Fukuda, 2019; Fischer-Tlustos et al., 2021). In this study, *Escherichia-Shigella*, a genus commonly associated with pathogenic microbes, was found to be higher in diarrheic calves at the time of disease. The lower relative abundance of this genus observed in healthy calves could be associated with the higher relative abundance of *Bifidobacterium*; however, after correlation analysis, only a weak negative association was observed between the two bacteria.

While it can be described as a commensal microbe in the GIT, *Peptostreptococcus* has pathogenic potential (Murdoch, 1998). It has been previously associated with calf diarrhea (Hennessy et al., 2021), gastrointestinal disease (Tang et al., 2023), and increasing antimicrobial resistance in humans (Tang et al., 2022). In this study, it was also associated with neonatal calf diarrhea, being higher in relative abundance at the time of disease in diarrheic calves. It could be suggested that dysbiosis that occurs in the gut prior to diarrheal disease may allow for this microbe to transit from beneficial to detrimental. However, in order to fully understand how *Peptostreptococcus* changes its role within the gut, further knowledge is needed regarding its function before and during disease. As *Peptostreptococcus* produces lactic acid, Gomez et al. (2022) similarly associated the increase in lactate-producing microbes with diarrheal disease at the time of disease manifestation. Increased lactic acid production results in acidemia, and increased anion gap acidosis, which has been associated with diarrheal disease (Gomez et al., 2022). The diarrheic calves in this study not only had a higher relative abundance of *Peptostreptococcus*, but also saw an increase in RA of *Ligilactobacillus* (Gomez et al., 2022) and *Faecalicoccus* (De Maesschalck et al., 2014) both known to be lactate producers.

Many of the microbes associated with health status in the current study have been identified in other studies focusing on the time of disease incident. However, the fecal microbiome of diarrheic calves at disease manifestation was dominated by the genus *Alloprevotella*. This genus is an obligate anaerobe (Downes et al., 2013), and its appearance in high relative abundance contradicts the findings by Gomez et al. (2022) that the fecal microbiomes of diarrheic calves are characterized by a shift from obligate to facultative anaerobes. In humans, it has been found as a normal gut microbe and has been shown to decrease in the relative abundance during disease (Tian et al., 2021). However, Tang et al. (2023) found that *Alloprevotella* was enriched in patients with irritable bowel syndrome and suggested that this microbe may produce a diarrheal incident by promoting the formation of certain short chain fatty acids and inflammation. In a study performed in mice, *Alloprevotella* was positively correlated with the production of tumor necrosis factor (TNF) α (Yang et al., 2021). TNF α is a powerful pro-inflammatory cytokine associated with immune response (Idriss and Naismith, 2000). This may suggest that the increased relative abundance of *Alloprevotella* in the fecal microbiota of diarrheic calves is linked to the inflammatory and immune response occurring at the mucosal level, but without further investigation through metatranscriptomics; the potential association between this genus and diarrheal disease in pre-weaned calves is unclear.

It is possible that the higher relative abundance of certain commensal microbes observed in diarrheic calves at disease manifestation may reflect the microbial community attempting to resolve the dysbiotic episode. To better understand the presence and function of these microbes at the time of disease, and their potential to become pathogenic, further investigation is required.

### 4.4 Illustrating the changing composition preceding diarrheal disease

No ASVs were associated with health status pre-diarrheal disease or post-disease. This suggests that prior to diarrheal disease manifestation, there were no differences in the fecal microbiota of calves, and that following diarrheal disease, the fecal microbiota were able to “recover” and re-establish an equilibrium. There were, however, changes in the relative abundance of certain ASVs that occurred in between sampling time-points were significantly associated with health status. Examining the progression of these ASVs alongside the correlations observed between them may allow for the characterization of the dysbiosis that occurs prior to diarrheal disease. This information may prove useful for the development of more targeted preventative measures and treatments at the time of disease, such as probiotics, prebiotics, and feed additives.

From pre-disease to disease manifestation, the relative abundance of *Bifidobacterium* in both cohorts increased and decreased as calves aged. This is expected as the calf transitions from a liquid diet to a solid-feed diet (Amin and Seifert, 2021). In diarrheic calves, the decrease appears to be more marked than healthy calves. *Escherichia-Shigella* has previously been observed at high abundance during the first week of life (Song et al., 2018). It has been suggested that as *Bifidobacterium* becomes more prevalent, it outcompetes *Escherichia-Shigella* (Picard et al., 2005; Aw and Fukuda, 2019). The weak negative correlation observed in the current study, however, suggests that this relationship is not as strong as it appears. Healthy calves did experience a steeper decline in the relative abundance of *Escherichia-Shigella* than diarrheic calves, and the inverse was true for *Bifidobacterium,* which supports the benefits of using *Bifidobacterium* preventatively as a probiotic, or even as a treatment at the time of disease, but also suggests that the proliferation of these two genera within the hindgut are not as closely linked as previously thought.

*Prevotella* is a part of the normal flora of the GIT and has been associated with healthy calves (Chen et al., 2022; Gomez et al., 2022). It has been suggested to be a part of the core microbiota during the pre-weaning period (Chen et al., 2022). In this study, however, *Prevotella* was present in higher relative abundance in diarrheic calves. Both cohorts started with a similar relative abundance, yet over the same time frame, a greater increase was observed in diarrheic calves than in healthy calves. A similar pattern was observed for *Alloprevotella*.

In a recent study carried out by Chen et al. (2022), examining the dynamics of the fecal microbiome over the pre-weaning period in *Clostridium_sensu_stricto_1* was suggested as a potential biomarker for risk of diarrheal disease. It was also negatively associated with various members of *Prevotella* and *Alloprevotella* genera. *Clostridium_sensu_stricto_1* was not significantly associated with health status in the current study, which may lend some explanation to the proliferation of *Alloprevotella* and *Prevotella* identified in the feces of calves. The findings of the current study do not support the suggestion of Chen et al. (2022) to use *Clostridium_sensu_stricto_1* as a biomarker for risk of diarrhea, but this does highlight the variations observed in the literature across farms and environments. With this in mind, the development of diagnostic tools using microbes as biomarkers to determine the risk of diarrheal disease would need to be tested or validated across breeds and environments or designed in a way that it detects the decline of known commensals that are common, regardless of environmental factors, which can be used as indicators of oncoming dysbiosis.

Upon examining the ASVs significantly associated with health status as the calf transitions from pre-disease to disease manifestation, what is notable is not necessarily an increase in the relative abundance of potentially pathogenic microbes, but the change in abundance of commensal microbes. Ma et al. (2020) suggest that dysbiosis is the result of diarrheal incidence; however, the findings in the current study suggest that diarrheal incidence is the result of fecal bacterial dysbiosis. The correlations observed between these ASVs at disease manifestation provide an insight into the way bacterial genera interact with one another, as dysbiosis and the reduction in microbial diversity occurs. The strong and moderate negative correlations observed between *Alloprevotella, Bifidobacterium, Faecalicoccus, and Erysipelatoclostridium* and unclassified *Ruminococcaceae* help depict the genera that were lost, which allowed the proliferation of other microbes that resulted in diarrhea. The loss or lack of abundance of *Bifidobacterium* is regularly associated with neonatal calf diarrhea (Gomez et al., 2017; Slanzon et al., 2022; Cui et al., 2023). The ASVs that were significantly positively correlated to this genus may provide insight into relationships between bacteria that may be beneficial to the development of the hindgut microbiome and the prevention of diarrhea. In a recent study by Cui et al. (2023), a reduction in *Bifidobacterium, Colinsella, Erysipelatoclostridium,* and *Faecalicoccus* was associated with viral diarrhea in neonatal calves. Ma et al. (2020) also found *Erysipelatoclostridium* to be a potential microbial marker for diarrheal disease, such that it appeared in higher relative abundance in healthy calves, which was also observed in the current study. The correlations observed in the current study support the findings by Cui et al. (2023) and Ma et al. (2020) and add strength to the potential relationships observed between these genera during pre-weaning development of the hindgut microbiome. Revealing the correlations between these genera could allow for the development of probiotics containing diverse communities of bacteria that could help prevent dysbiosis and the resulting diarrheal disease.

## 5 Conclusion

Diarrheal disease is complex, multifactorial and is associated with dysbiosis of the GIT microbiome. The factors that influence the development of the GIT microbiome are equally complex and multifactorial. To better understand the different facets of diarrheal disease and microbiome development, it was essential to analyze fecal samples collected during the pre-weaning period. In this study, colostrum source and calf breed had no effect on the composition of the fecal microbiome in healthy and diarrheic home bred calves. Time-point and health status and the interaction between the two did significantly impact the composition and the progression of the fecal microbiota. Examination of microbes associated with health status before, during, and after disease created a visualization of the dysbiosis that may occur leading to diarrheal incident. Correlations between ASVs at the time of diarrhea demonstrated the loss of commensal bacteria in the fecal microbiome, which resulted in dysbiosis and lead to diarrheal disease. However, investigation of the functional role of these microbes within the gut before, during, and after disease is required potentially through metatranscriptomics. The decreased microbial diversity at disease manifestation, the correlations between genera, and significant changes in the abundance of commensal microbes leading to disease may highlight the need to focus not only on pathogenic microbes within the microbiome but also the commensals and the associated changes within these communities. This information may allow for the development of better diagnostic and preventative tools and potentially alternative treatments for diarrheal disease.

## Data availability statement

The datasets presented in this study can be found in online repositories. Additional supplementary material can be accessed through Open Science Framework at: https://doi.org/10.17605/OSF.IO/U624N. The names of the repository/repositories and accession number(s) can be found at: https://www.ncbi.nlm.nih.gov/, PRJNA1018835.

## Ethics statement

The animal studies were approved by the Teagasc Animal Ethics Committee (TAEC2021-327). All animal procedures used in the study and conducted using procedures consistent with the experimental license (AE19132/P148) issued by the Irish Health Products Regulatory Authority in accordance with European Union legislation (Directive 2010/63/EU) for the protection of animals used for scientific purposes. The studies were conducted in accordance with the local legislation and institutional requirements. Written informed consent was obtained from the owners for the participation of their animals in this study.

## Author contributions

SS: Writing – review & editing, Writing – original draft, Methodology, Investigation, Formal analysis, Data curation, Conceptualization. BE: Writing – review & editing, Writing – original draft, Supervision, Project administration, Methodology, Investigation, Formal analysis, Data curation, Conceptualization. PS: Writing – review & editing, Writing – original draft, Methodology, Formal analysis, Data curation, Conceptualization. CM: Writing – review & editing, Writing – original draft, Supervision, Methodology, Formal analysis, Data curation. SW: Writing – review & editing, Writing – original draft, Supervision, Resources, Project administration, Methodology, Investigation, Funding acquisition, Formal analysis, Data curation, Conceptualization.

## Footnotes

^1^ https://benjjneb.github.io/dada2/training.html
